# Examining the Relationship between Urogenital Schistosomiasis and HIV Infection

**DOI:** 10.1371/journal.pntd.0001396

**Published:** 2011-12-06

**Authors:** Pamela Sabina Mbabazi, Olivia Andan, Daniel W. Fitzgerald, Lester Chitsulo, Dirk Engels, Jennifer A. Downs

**Affiliations:** 1 Department of Control of Neglected Tropical Diseases, World Health Organization, Geneva, Switzerland; 2 Center for Global Health, Division of Infectious Diseases, Weill Cornell Medical College, New York, New York, United States of America; Hospital Universitário, Brazil

## Abstract

**Background:**

Urogenital schistosomiasis, caused by infection with *Schistosoma haematobium*, is widespread and causes substantial morbidity on the African continent. The infection has been suggested as an unrecognized risk factor for incident HIV infection. Current guidelines recommend preventive chemotherapy, using praziquantel as a public health tool, to avert morbidity due to schistosomiasis. In individuals of reproductive age, urogenital schistosomiasis remains highly prevalent and, likely, underdiagnosed. This comprehensive literature review was undertaken to examine the evidence for a cause-effect relationship between urogenital schistosomiasis and HIV/AIDS. The review aims to support discussions of urogenital schistosomiasis as a neglected yet urgent public health challenge.

**Methodology/Principal Findings:**

We conducted a systematic search of the literature including online databases, clinical guidelines, and current medical textbooks. We describe plausible local and systemic mechanisms by which *Schistosoma haematobium* infection could increase the risk of HIV acquisition in both women and men. We also detail the effects of *S. haematobium* infection on the progression and transmissibility of HIV in co-infected individuals. We briefly summarize available evidence on the immunomodulatory effects of chronic schistosomiasis and the implications this might have for populations at high risk of both schistosomiasis and HIV.

**Conclusions/Significance:**

Studies support the hypothesis that urogenital schistosomiasis in women and men constitutes a significant risk factor for HIV acquisition due both to local genital tract and global immunological effects. In those who become HIV-infected, schistosomal co-infection may accelerate HIV disease progression and facilitate viral transmission to sexual partners. Establishing effective prevention strategies using praziquantel, including better definition of treatment age, duration, and frequency of treatment for urogenital schistosomiasis, is an important public health priority. Our findings call attention to this pressing yet neglected public health issue and the potential added benefit of scaling up coverage of schistosomal treatment for populations in whom HIV infection is prevalent.

## Author Summary

Urogenital schistosomiasis is a parasitic infection caused by a worm, *Schistosoma haematobium*, which lives in the bloodstream of infected individuals. It affects at least 112 million people, mostly in sub-Saharan Africa, and has been suggested to be a risk factor for becoming infected with HIV. We reviewed publications in order to examine whether it seems likely that this parasitic infection could be a risk factor for HIV. Evidence from many types of studies supports the hypothesis that urogenital schistosomiasis does increase a person's risk of becoming infected with HIV. Studies also suggest that individuals who have both urogenital schistosomiasis and HIV have a more aggressive HIV infection and can more easily transmit HIV to their sexual partners. Praziquantel is an oral, nontoxic, inexpensive medication that is safe in pregnancy and is recommended for treatment of schistosomiasis. In areas where both infections co-exist, regular administration of praziquantel both to young girls and to sexually-active women may be an important approach to reducing HIV transmission. Our findings support the importance of making praziquantel more available to people who live in areas of the world where both urogenital schistosomiasis and HIV infection are widespread.

## Introduction

An estimated 207 million people worldwide are infected with schistosomes [1], and 85% of these cases occur in Africa [1]–[3]. Schistosomiasis is a disease of poverty that arises in areas with poor sanitation where people come into contact with urine- or feces-contaminated water as part of their daily lives [4]. Individuals living in endemic countries are most commonly infected during childhood, and the prevalence peaks between the ages of 10 and 20 years [5], [6]. For those who are continually reinfected by contaminated water, schistosomiasis causes a chronic disease over decades. While the mortality caused by schistosomiasis is low, the morbidity is high, and includes anemia, stunted growth, and decreased ability to learn in children [1]. For these reasons, the World Health Organization (WHO) recommends annual treatment for school-aged children in areas of high endemicity [7].

*Schistosoma haematobium* causes more than half (at least 112 million) of worldwide schistosome infections [8]. Formerly known as urinary schistosomiasis, *S. haematobium* infection was recently renamed “urogenital schistosomiasis” in recognition that the disease affects both the urinary and genital tracts in up to 75% of infected individuals [9]. Adult *S. haematobium* worms inhabit the venules surrounding organs of the pelvis where they lay between 20 and 200 eggs daily [4]. These eggs subsequently penetrate the vessel wall and move towards the lumen of the bladder. An important proportion of the eggs become sequestered in the tissue of pelvic organs such as the urinary bladder, lower ureters, cervix, vagina, prostate gland, and seminal vesicles, where they cause chronic inflammation in the affected organs. This results in a number of symptoms and signs including pelvic pain, postcoital bleeding, and an altered cervical epithelium in women [10]–[11], and ejaculatory pain, hematospermia and leukocytospermia in men [12]–[13].

**Figure 1:**
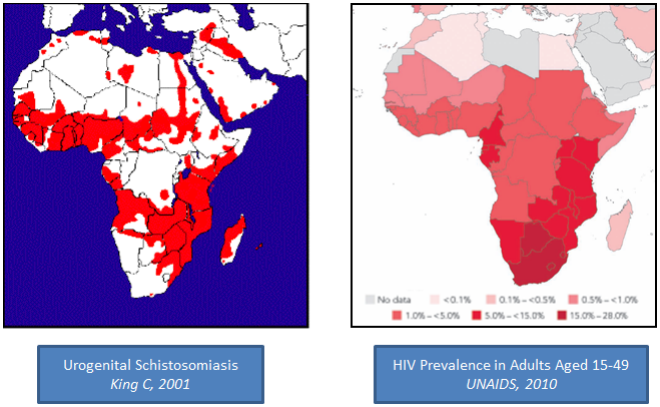
Geographical overlap of *S. haematobium* and HIV infections [**14]**-[15****].

Epidemiologic mapping studies of HIV and *S. haematobium* in Africa depict a substantial overlap, in many regions, between areas in which *S. haematobium* is endemic and areas in which women have a high prevalence of HIV infection (**Figure 1**) [14]. In particular, HIV studies report an unexplained gender quotient disfavoring women over men [15]–[16]. In rural women whose limited access to clean water more often puts them at risk for schistosomiasis, HIV prevalence also peaks at younger ages than in urban women [17]. While some of this skewing has been attributed to social, behavioral, and cultural norms [17]–[20], this unexplained gender quotient also suggests that risk factors for HIV acquisition may be different between rural and urban populations [17].

Several cross-sectional studies have reported associations between urogenital schistosomiasis and HIV, but the infection still receives relatively little attention. Global Burden of Disease (e.g. disability-adjusted life years [DALY]) calculations treat schistosomiasis cases as a single sequel, leading others to argue that the estimates should be much higher [21]–[23]. Furthermore, DALY calculations have neither examined urogenital schistosomiasis as an entity separate from intestinal schistosomiasis nor considered it as a population-attributable risk factor for HIV transmission. Thus urogenital schistosomiasis remains a neglected disease, particularly in women and men of reproductive age.

This comprehensive literature review was undertaken to examine the evidence for a cause-effect relationship between urogenital schistosomiasis and HIV/AIDS. Our aim is to support discussions of urogenital schistosomiasis as an urgent public health challenge [24].

## Methods

We conducted a broad review of the literature by performing a systematic search of online databases including PUBMED/MEDINE, EMBASE, POPLINE, GLOBAL HEALTH, and WEB OF KNOWLEDGE. We used search terms beginning with the text string ‘schistosom’ in all possible combinations with ‘HIV’, ‘HIV/AIDS’, and other related keywords including ‘urinary,’ ‘genital’, ‘gynecology’, and ‘adolescent.’ Case reports were excluded. We subsequently limited our search to articles published in the past 30 years which overlap with the HIV pandemic.

We also reviewed the most current editions of widely-used infectious diseases textbooks [6], [25]–[28] and WHO websites for relevant publications. We screened titles and abstracts for relevance, and subsequently reviewed the full texts of manuscripts that were potentially pertinent. Notable manuscripts and key learning points that emerged during our review are summarized in Tables 1 and 2.

**Table 1:** Five key papers in the field.

1	Kjetland EF, Ndhlovu PD, Gomo E, Mduluza T, Midzi N, et al (2006) Association between genital schistosomiasis and HIV in rural Zimbabwean women. *AIDS* 20(4): 593–600.
2	Leutscher PDC, Pedersen M, Raharisolo C, Jensen JS, Hoffmann S, et al (2005) Increased prevalence of leukocytes and elevated cytokine levels in semen from *Schistosoma haematobium*-infected individuals. *J Infect Dis* 191: 1639–47.
3	Secor WE (2006) Interactions between schistosomiasis and HIV-1. *Parasit Immunol* 28: 597–603.
4	Kallestrup P, Zinyama R, Gomo E, Butterworth AE, Mudenge B, et al (2005) Schistosomiasis and HIV-1 infection in rural Zimbabwe: effect of treatment of schistosomiasis on CD4 count and plasma HIV-1 RNA load. *J Infect Dis* 192(11): 1956–61.
5	Poggensee G and Feldmeier H (2001) Female genital schistosomiasis: facts and hypotheses. *Acta Tropica* 79: 193–210.

**Table 2:** Key learning points.

1	*Schistosoma haematobium* infection has been recently renamed “urogenital schistosomiasis” by the World Health Organization because of its tissue-damaging effects of both the urinary and genital tracts.
2	*S. haematobium* ova in the female genital tract (typically the cervix and vagina) cause epithelial inflammation leading to erosions and ulcerations. These epithelial breaches may facilitate HIV viral entry and binding to immune cells present in the altered epithelium.
3	In men with *S. haematobium* infection, genital organs and semen show inflammatory alterations that may imply an increased risk of HIV transmission from men to women.
4	Chronic schistosomiasis promotes a Th2-type immune environment in the host that may increase susceptibility to HIV infection and viral propagation.
5	In HIV-positive individuals, co-infection with schistosomes appears to raise HIV RNA levels and decrease CD4+ T-cell counts more rapidly than in those without co-infection. This could lead to a more rapid disease progression and a higher viral load which, in turn, enhances virus shedding through genital secretions.

## Results and Discussion

### Local Effects of Urogenital Schistosomiasis and Susceptibility to HIV Infection in Women

#### Macroscopic Tissue Damage

Reported symptoms of female genital schistosomiasis include infertility, pelvic discomfort, dyspareunia, contact and spontaneous bleeding, itching, and giant granulomata that appear as tumors [10]–[11], [29]–[30]. The condition has been associated with mucosal edema, erosion and ulcerations leading to a friable epithelium [10], most commonly on the cervix [29]. Similar breaches in the integrity of the genital epithelium, whether due to trauma or to sexually-transmitted ulcerative diseases, have been associated with an increased risk of HIV infection. The epithelial damage caused by *S. haematobium* infection, which has been observed to persist even after treatment, has been suggested to ease viral entry and therefore could serve as a facilitating factor for acquisition of HIV [11], [31]–[32]. Schistosomiasis in the female genital tract has been postulated to pose a greater risk than bacterial genital ulcer disease because, unlike many common sexually-transmitted infections (STIs), it often is not restricted to a single localized sore that allows the rest of the vulval, vaginal or cervical epithelium to remain intact [33].

As they can become sequestered in any genital organ, schistosomal ova cause a spectrum of clinical pathology. “Grainy sandy patches,” which are tiny cervical abnormalities similar to sandy patches in the bladder, have been recognized as pathognomonic for female genital schistosomiasis [10]. Grainy sandy patches have been described as rough small grains located superficially within the mucosa [10]. A second type of sandy patch, the “homogeneous yellow sandy patch,” has also been associated with *S. haematobium* infection but is less specific since it is also associated with STIs [10]. Grainy sandy patches are often encircled by irregularly-formed blood vessels believed to represent egg-induced neovascularization [10], [34]. This may explain the contact bleeding that is associated with genital tract *S. haematobium* infection and could be one mechanism by which HIV risk is enhanced.

#### Microscopic Tissue Damage

Sequestered schistosomal ova have been shown to evoke a complex cellular and humoral immune response in tissue. The ova of *S. haematobium* can induce areas of inflammation or of large granulomata in the female genital tract, with recruitment of plasma cells, lymphocytes, granulocytes, macrophages, and eosinophils to the site [35]–[36]. These inflammatory cells express CD4+ T-cell receptors, which are the primary targets for HIV. This activity at cellular level in genital lesions caused by schistosomiasis is potentially comparable to lesions caused by primary and secondary syphilis and herpes simplex virus infections (HSV-1 and HSV-2), in which affected tissue was shown to have more HIV receptors than healthy tissue nearby [37]. Monocytes and CD4+ T-cells in individuals infected with *S. mansoni* have been reported to display higher surface densities of *Schistosoma*-induced chemokine receptors CCR5 and CXCR4 [38].

Inflammation provoked by ova in genital tissue has thus been postulated to recruit these activated immune cells expressing CD4, CXCR4, and CCR5 receptors into the epithelium, facilitating rapid binding of the virus after penetration through an ulcerated, friable epithelium [39]. In support of this hypothesis, rhesus macaques with acute *S. mansoni* infection were found to be more susceptible to development of systemic HIV infection after rectal HIV exposure than were macaques without *S. mansoni* [40]. Because macaques did not have statistically-significant differences in the rates of HIV infection after intravenous HIV exposure, the authors concluded that the increased host susceptibility to HIV in the setting of schistosomal infection that they observed was predominantly due to mucosal inflammation [40].

#### Findings from Clinico-Epidemiological Studies

A few cross-sectional studies in women support an association between urogenital schistosomiasis and HIV infection (Table 3). In Zimbabwe, women with urinary schistosomiasis had a higher HIV prevalence (33.3%) than women without urinary schistosomiasis (HIV prevalence of 25.6%, p = 0.053) [41]. Women with *S. haematobium* ova in the genital tract had nearly a three-fold increased risk of having HIV [34]. In Tanzania, women with *S. haematobium* infection had a four-fold increased risk of HIV [42].

**Table 3:** Primary studies in individuals with schistosomiasis and HIV infection.

ARTICLE	MAJOR FINDING	LIMITATIONS
**Epidemiological Studies**		
Ndhlovu P et al, *Trans RoySoc Trop Med Hyg* 2007 [41]	Women in Zimbabwe with urinary schistosomiasis had a higher prevalence of HIV than those without urinary schistosomiasis(33% vs. 26%, p = 0.053).	Cross-sectional studies and thereforeunable to demonstrate causality.
Kjetland EF et al, *AIDS*2006 [32]	HIV was associated with female genital schistosomiasis inZimbabwean women (OR = 2.9, 95% CI [1.1–7.5]).	
Downs JA et al, *Am J TropMed Hyg* 2011 [42]	HIV was associated with *S. haematobium* infection in Tanzanianwomen (OR = 4.0 [1.2–13.5]).	
**Immunological Studies in Individuals with HIV/** ***S. mansoni*** ** Co-Infection**		
Mwinzi PN et al, *J Infect Dis*2001 [56]	*S. mansoni*-infected individuals who were also HIV-positive hadlower levels of Th2-type cytokines than those without HIV,implying HIV-mediated Th2-type CD4+ T-cell descruction.	Unable to demonstrate definitively that lowercytokine levels reflect Th2-type cell destructionby HIV rather than being caused by another mechanism.
Secor WE et al, *InfectImmunol* 2003 [38]	Densities of the chemokine receptors CCR5 and CXCR4 on CD4+T-cells in HIV-positive (and also HIV-negative) individuals with *S.mansoni* co-infection were reduced following praziquantel treatment.	Very small observational study; possibilityof selection bias not addressed.
**Observational Studies Assessing Effect of Praziquantel Treatment for ** ***S. mansoni*** ** on HIV-RNA levels in Individuals with HIV Infection**		
Lawn SD et al, *AIDS*2000 [62]	HIV RNA levels increased significantly over a mean of 5.6 monthsof follow-up.	Observational studies: -Effects may be moreattributable to length of follow-up time and toeffects of HIV infection than to praziquantel treatment.-No control groups or randomization.
Elliott AM et al, *Trans RoySoc Trop Med Hyg* 2003 [63]	HIV RNA levels transiently increased (at 5 weeks after treatment)and then returned to pre-treatment baseline by 4 months.	
Brown M et al, *J Infect Dis*2004 [64]	HIV RNA levels were not significantly different pre- andpost- treatment in HIV/*S. mansoni* co-infected individuals.	
Modjarrad K et al, *J InfectDis* 2005 [65]	HIV RNA levels increased (nonsignificantly) over the 16-weekpost-treatment follow-up.	
Brown M et al, *J InfectDis* 2005 [66]	HIV RNA levels transiently increased 1 month after treatmentand then returned to pre-treatment levels by month 5.	
**Randomized Trial Assessing Effect of Praziquantel Treatment for ** ***S. mansoni*** ** in Individuals with HIV Infection**		
Kallestrup P et al, *J InfectDis* 2005 [60]	HIV-positive patients who were randomized to receivepraziquantel immediately had smaller HIV RNA level increasesand increased CD4+ T-cell count compared with thoserandomized to treatment after 3 months.	Randomized but not blinded so potentialfor bias in follow-up.

Taken together, these studies included more than 1000 African women from rural communities with the consistent finding that *S. haematobium* infection was associated with HIV, with odds ratios between 2.9 and 4.0. Each of these studies is limited by its cross-sectional nature, which allows determination only of an association, rather than of a cause-effect relationship, between *S. haematobium* infection and HIV. Because *S. haematobium* infection, typically acquired in childhood, normally precedes HIV acquisition, it seems likely that *S. haematobium* infection may increase the susceptibility to HIV infection when girls reach sexual maturity. Definitive proof of this hypothesis can only be ascertained through longitudinal studies that are carefully planned to demonstrate a cause-effect relationship.

The design of prospective studies to establish a causal relationship would be complex and needs to address difficult ethical issues concerning gynecological examination of pre-teenage and teenage girls. Moreover, withholding praziquantel from young women with documented *S. haematobium* infection and following them prospectively as a control group in a study for incident HIV infections is not ethical. For these reasons, recent study designs have focused on comparing different mass treatment strategies among cohorts of girls at different ages in *S. haematobium-*endemic communities. For example, young adolescent schoolgirls would receive early, regular praziquantel prophylaxis and be compared, at the time they commence sexual activity, to older adolescent girls who received fewer treatments before becoming sexually-active and to older adolescent “control” girls from the same villages who are sexually active and did not receive prophylaxis. The hypothesis is that early, frequent praziquantel treatment will prevent development of genital lesions in adolescence and will consequently reduce HIV infections in girls and women after sexual debut [14], [43]. Another possibility would be to use recently-developed serum schistosomal antigen tests, such as the Circulating Anodic Antigen (CAA) [44], [45], to analyze banked serum from prior HIV seroincidence studies. These study designs also highlight additional research needs in the field, such as optimization of the praziquantel regimen for treatment of urogenital schistosomiasis and the need for a standardized, agreed-upon case definition for clinical diagnosis and measurement of morbidity caused by urogenital schistosomiasis.

### Female Genital Schistosomiasis and Shedding of HIV

Concurrent *S. haematobium* infection may also increase the ease with which HIV-positive women transmit HIV infection to their sexual partners. A recent meta-analysis found that a variety of genital tract infections were associated with HIV-1 viral shedding in the female genital tract [46]. This effect was most pronounced in conditions that resulted in the recruitment of high concentrations of leukocytes to the genital epithelium, including nonspecific cervicitis, genital ulcer diseases, *Chlamydia trachomatis* and *Neisseria gonorrhea* infections, and vulvovaginal candidiasis. The authors hypothesized that, because leukocytes typically harbor HIV, conditions that lead to higher genital tract leukocyte concentrations are those that most heighten the risk of sexual HIV transmission from women to men during sexual intercourse. Given the recruitment of leukocytes to the genital tract by *S. haematobium* infection [35], [36], it seems possible that, through this mechanism, women who are co-infected with HIV and *S. haematobium* may more easily transmit HIV to their sexual partners.

### Male Urogenital Schistosomiasis and HIV Infection

Genital schistosomiasis in men can involve several male reproductive organs. Since the penis is not affected by ova-induced lesions, male genital schistosomiasis is not believed to increase the risk of HIV acquisition through local effects [43] but rather through schistosomiasis-related immunomodulatory effects. Moreover, the infection could increase risk for HIV transmission by inciting inflammation in the male genital tract. Men with severe urogenital schistosomiasis have been found to have a higher prevalence of lymphocytes and eosinophils in seminal fluid than those without infection [47]. Infected men are also reported to have significantly higher levels of interleukin (IL)-4, IL-6, IL-10, and tumor necrosis factor-alpha in their semen. These cytokines may recruit more HIV-infected cells to the semen, upregulate viral replication, and increase the concentration of HIV virus in semen [47]. Six months after anti-schistosomal treatment, the concentrations of seminal lymphocytes and eosinophils were lower and the levels of cytokines were reduced. Another analysis of seminal fluid in *S. haematobium*-infected men demonstrated lower volumes of semen, higher levels of eosinophilic cationic protein (an established marker of inflammation and morbidity in urogenital schistosomiasis), and higher rates of sperm apoptosis, which lessened after praziquantel treatment [48].

With a mechanism similar to that discussed for women in the preceding section, it has been demonstrated that HIV-positive men with concomitant genital tract infections, such as urethritis, have higher concentrations of seminal HIV-1 RNA than those without dual infections [49]. The chronic inflammation and recruitment of lymphocytes and eosinophils to the male genital tract may increase the HIV-1 viral load in semen. In this manner, a female sexual partner of an HIV-positive male living in an *S. haematobium*-endemic area may have a doubly-amplified risk of HIV acquisition: her *S. haematobium-*infected partner's semen may contain disproportionately high concentrations of HIV RNA, and her own *S. haematobium* infection may increase the ease with which HIV can establish infection following exposure. Taken together, these data suggest that egg-induced inflammation in the male genital tract could be a risk factor for HIV transmission from men to women.

### Schistosomiasis in Children and Adolescents and HIV Transmission

Schistosomal lesions are commoner in the vulva and the lower vagina before puberty, while in adult women they are more frequent in the cervix, uterus, ovaries and fallopian tubes [50]. These clinically-apparent lesions and the resulting compromise of the vaginal epithelium, therefore, are already present before a girl's first sexual intercourse. This is in contrast to lesions caused by STIs, which can develop only after sexual intercourse [32]. The presence of schistosomal lesions already in childhood makes it likely that schistosomal infection typically precedes HIV infection and that the temporal association reflects the fact that urogenital schistosomiasis is a risk factor for HIV acquisition rather than vice-versa [10].

Important risk factors for urinary tract morbidity in adulthood are cumulative intensity and duration of *S. haematobium* infection during early adolescence. Treatment of school-aged children can significantly reduce the cumulative lifetime egg burden as the intensity of infection is greatest during early teenage years [50]. Furthermore, treatment for schistosomiasis during childhood was significantly associated with the absence of cervical sandy patches and contact bleeding in adult women [51]. Thus treatment of *S. haematobium* infection before and during the teenage years may not only diminish genital schistosomiasis-associated morbidity in adulthood, but may simultaneously decrease the risk of HIV acquisition.

### Schistosomiasis-Associated Immunomodulation and the Risk of HIV Infection

In addition to local and gender-specific effects of *S. haematobium* infection, schistosomiasis also appears to increase HIV susceptibility through chronic immune modulation. This topic has been studied far more extensively with regard to *Schistosoma mansoni* [52]. It has also recently been the subject of a comprehensive review [31] and for this reason will be summarized only briefly here.

Chronic schistosomiasis alters global immune function and in this manner may also increase susceptibility to HIV infection [38]. It preferentially stimulates the Th2-type immune response, with reciprocal down-regulation of the Th1-type cytotoxic responses [53] which are important in initial control of HIV infection. This is supported by work from Uganda, which demonstrated that HIV-positive patients with *S. mansoni* infection had decreased Gag-specific cytolytic CD8+ responses [54]. Moreover, CD4+ T-cells with a Th2 phenotype are more readily infected, and subsequently destroyed, by HIV-1 than are Th1 cells [55]. In individuals in Kenya with HIV and *S. mansoni* co-infection, Th2-type CD4+ T-cells were destroyed more quickly than in HIV-positive individuals without schistosomiasis [56].

Specifically, differences in cell surface receptors may lead to differences in HIV susceptibility between those with and without schistosomiasis. The chemokine receptors CCR5 and CXCR4 are co-receptors for HIV-1 and were found to be more dense on the CD4+ T-cell surfaces of individuals with active *S. mansoni* infection than on the CD4+ T-cells of individuals who had received prior anti-schistosomal treatment [38]. The levels of these co-receptors dropped in individuals who were studied pre- and post-praziquantel treatment [38]. This highlights the potential role that widespread anti-schistosomal treatment could play in reducing the progression and spread of HIV.

### Effects of Schistosomiasis on Progression of HIV Infection and Shedding of HIV

In addition to potentially increasing susceptibility to HIV infection, evidence suggests that *S. haematobium* infection may also speed progression of disease by raising plasma HIV RNA concentration (commonly known as “viral load”) in individuals who are co-infected. At the cellular level, the same CCR5 and CXCR4 chemokine receptors that are upregulated in schistosomiasis and facilitate HIV binding in initial infection may also promote cell-to-cell spread of HIV once infection is established [31], [57]. Multiple studies have shown that the plasma HIV RNA level is predictive of both HIV disease progression and risk of transmission of HIV to sexual partners [58], [59]. If the hypothesis is correct that schistosomiasis increases the HIV RNA levels in co-infected individuals, then treatment for schistosomiasis could delay the development of AIDS and decrease the spread of HIV in sub-Saharan Africa.

A recent randomized clinical trial conducted in Zimbabwe supports this hypothesis. Patients who were infected with both HIV and *S. mansoni* were randomized either to praziquantel treatment at enrollment or to praziquantel after three months [60]. Compared with the group in whom treatment was delayed, the early-treatment group experienced significantly smaller declines in CD4+ T-cell counts after three months (mean decline of 1.7 cells/ µL versus 35.2 cells/ µL in the delayed-treatment group) [61]. Notably, the HIV RNA levels in both groups of patients increased during the three months, but the mean increase in the early-treatment group (0.001log_10_ copies/mL) was significantly lower than in the delayed-treatment group (0.21log_10_ copies/mL).

Earlier non-randomized studies of HIV-positive patients who were treated for *S. mansoni* infections had found that HIV RNA levels remained stable or increased in patients regardless of treatment [62]–[65]. One notable study that reported significant HIV RNA level increases one month post-treatment noted corresponding increases in *S. mansoni-*specific Th2-type cytokine responses as well, though both of these reverted to pre-treatment levels by five months post-treatment [66]. Notably, none of the patients in these studies were receiving ART. In light of the findings of the randomized trial in Zimbabwe that did demonstrate a benefit with praziquantel treatment with regard to the viral load [60], it is plausible that the overall observed increases in HIV RNA levels reflect natural progression of untreated HIV infection. In this sense, while treatment for schistosomiasis is clearly not able to substitute for antiretroviral therapy, it may possibly be able to slow HIV disease progression [31].

In support of this hypothesis, two other randomized studies of HIV-infected patients co-infected with either *Wucheria bancrofti* [67] or soil-transmitted helminths [68] have explored the effects of treatment on parameters of HIV infection. Patients treated for lymphatic filariasis had significant decreases in their HIV RNA levels and insignificant increases in their CD4+ T-cell counts at 12 weeks as compared to pretreatment levels [67]. Patients with ascariasis who received albendazole experienced significantly higher CD4+ T-cell counts at 12 weeks and a trend towards lower HIV RNA levels [68].

Taken together, these studies of treatment in HIV and helminth co-infections support a positive effect of antiparasitic treatment on certain HIV infection parameters. While treatment for schistosomiasis in HIV-positive patients may not decrease HIV RNA levels, it may slow the increase of viral levels. It is also possible that praziquantel treatment may enhance immunocompetence by promoting an increase in CD4+ T-cell counts and an increased NK cell function [69]–[71].

### Conclusions

*Schistosoma haematobium* infection is highly prevalent in sub-Saharan Africa. Increasing evidence supports that it is a plausible risk factor for HIV acquisition due both to its local genital tract effects in women, and to its chronic immunomodulatory effects in both men and women. It also could facilitate HIV transmission to the sexual partners of HIV-positive individuals with schistosomal co-infection, and could enhance HIV disease progression. Circumstantial, biological, immunological, and epidemiological evidence is strongly suggestive of a cause-effect relationship between *S. haematobium* and HIV infection.

Our review highlights the need for further innovative research, particularly appropriately-designed longitudinal studies which ultimately would be able to confirm the suggested causality of schistosomiasis in incident HIV infections. Such studies must carefully balance the ethical obligation to ensure treatment for study subjects while simultaneously managing to explore the cause-effect relationship between the two infections. This is by no means an easy task. Consideration should therefore be given to harnessing latent operational research opportunities that exist within the context of ongoing schistosomiasis control programs. These include studies such as exploring the effect of early, regular anti-schistosomal treatment of girls to prevent development of urogenital lesions in adolescence or testing for markers of active schistosomiasis in blood collected from HIV-positive women before their HIV-seroconversion.

Meanwhile, in schistosomiasis-endemic areas where coverage for preventive chemotherapy with praziquantel remains low, millions of individuals may be at higher risk for HIV infection. The presumptive causal association with HIV infection notwithstanding, urogenital schistosomiasis by itself leads to significant morbidity that can be lessened with inexpensive preventive chemotherapy. At an annual cost of about 40 cents per person [72]–[73], praziquantel stands as a powerful and economical public health intervention with the potential to prevent the development of urogenital lesions, prolong survival, and decrease new HIV infections on the African continent. In view of the plausible association between urogenital schistosomiasis and HIV transmission in areas where these infections are co-endemic, a salient effect on the health of millions of individuals could presumably be achieved if antischistosomal treatment and HIV prevention interventions were integrated. The WHO-recommended policy of early regular treatment of school-age children with praziquantel needs to be extended to adults and prioritized in national programs as a possible means of further preventing HIV infections in sub-Saharan Africa.
